# Self-reported changes in aggressive driving within the past five years, and during COVID-19

**DOI:** 10.1371/journal.pone.0272422

**Published:** 2022-08-01

**Authors:** Amanda N. Stephens, Steven Trawley, Justin Ispanovic, Sophie Lowrie

**Affiliations:** 1 Monash University Accident Research Centre, Clayton, Victoria, Australia; 2 Cairnmillar Institute, Melbourne, Victoria, Australia; Shahrood University of Technology, ISLAMIC REPUBLIC OF IRAN

## Abstract

Aggressive driving is a significant road safety problem and is likely to get worse as the situations that provoke aggression become more prevalent in the road network (e.g. as traffic volumes and density increase and the grey fleet expands). In addition, driver frustration and stress, also recognised as triggers for aggression, are likely to stay high because of the COVID-19 pandemic and associated burdens, leading to increased aggression. However, although drivers report that other drivers are becoming more aggressive, self-report data suggests that the prevalence of aggression has not changed over time. This may be due to the methods used to define and measure aggression. This study sought to clarify whether self-reported aggression has increased over a five-year period and across three different types of aggression: verbal aggression, aggressive use of the vehicle and personal physical aggression. The influence of COVID-19 lockdowns on own and others’ driving styles was also investigated. A total of 774 drivers (males = 66.5%, mean age = 48.7; SD = 13.9) who had been licensed for at least five years (M = 30.6, SD = 14.3), responded to an online survey and provided retrospective frequencies for their current aggression (considered pre-COVID-19 lockdowns) and five years prior. Two open ended questions were included to understand perceived changes in driving styles (own and others) during the COVID-19 pandemic. One third (33%) of drivers believed they were more aggressive now than five years ago but 61% of the sample believed other drivers were more aggressive now than five years ago. Logistic regression analyses on changes in self-reported aggression (same or decreased vs increased) showed the main factor associated with increases in aggressive driving was the perception that other drivers’ aggression had increased. Further, almost half the sample (47%) reported that other drivers had become riskier and more dangerous during, and soon after, the COVID-19 lockdowns. These results show that the driving environment is seen as becoming more aggressive, both gradually and as a direct result of COVID-19 lockdowns. The data indicate that this perceived increase in aggression is likely to provoke higher levels of aggression in some drivers. Campaigns to reduce aggression on the roads need to focus on changing road culture and improving interactions, or perceived interactions, among road users.

## Introduction

### 1.1 Aggressive driving is a road safety risk, and we need to know if this is increasing

Aggressive driving is a recognised road safety problem. Naturalistic driving studies have shown that aggressive speeding, tailgating or signal violations increase the odds of crash up to 15-fold compared to compliant driving [1]. While aggression can be multifaceted, it is generally grouped into three classifications of behaviour [2]. These are minor aggression, such as swearing, gesticulating or sounding the horn; aggressive violations, where the driver uses their vehicle aggressively (e.g. tailgating, speeding, signal violations, weaving in and out of traffic, etc); and road rage, extreme violence involving or attempting assault on another road user or their vehicle. Not surprisingly, the prevalence of these is inversely related to the severity; with minor aggressions being the most common and reported by the majority of drivers (i.e., around 70%) and road rage being infrequent reported by approximately 4% of drivers [2].

A common misconception among drivers is that only extreme aggression poses road safety risks. Indeed, risky vehicle use when angry such as speeding, tailgating, dangerous overtaking or running red lights increases crash risk significantly [1]. Therefore, road safety efforts continue to focus on such behaviours through education and enforcement campaigns. Likewise, the risks associated with road rage are clear. This can end with vehicle damage or intended or actual physical harm, the psychological repercussions of which may last a long time. However, the risk from minor aggression is often overlooked as a safety concern. Maintaining anger can increase the odds of crash involvement 10-fold [1], or lead to cognitive distractions and ruminations that jeopardise the ability to drive safely [3]. Simply put, while only a small percentage of drivers engage in behaviours that obviously increase crash risk, most drivers may unintentionally put themselves at greater risk of crash when angry and expressing this through minor aggression. It is therefore important to understand the factors underpinning this aggression to support drivers to reduce these behaviours.

A considerable amount of research has been undertaken to understand causes and consequences for driver aggression. Researchers have investigated different aspects of aggression ranging from driver characteristics, traits and motivations [4–7], driver anger [8,9], situations conducive to aggression [10–12], workplace aggressive driving [13,14] and potential interventions to reduce aggressive driving [15,16]. These studies have highlighted the complex interactions between driver characteristics and the situations that lead to anger and aggressive displays of that anger, in turn increasing crash risk.

However, less research has been undertaken to understand whether aggressive driving is increasing, and if so, why? This is important to know because globally, the road networks, and driving fleets are changing. For example, in Australia the number of registered vehicles on the road network is increasing [17], while the infrastructure and alternative transport options are not updating at the same pace. This means that travel congestion–a recognised trigger for anger and aggression [10,12,18]—is increasing, leading to more opportunities for frustration and anger. Characteristics of the driving fleet are also changing with more commercial drivers, a larger mobile workforce and advances in technology [17] all of which are related to anger and aggression experiences. This means that a greater percentage of the fleet will be driving more often and covering longer distances, thus providing more exposure to anger provoking and frustrating events. There is also more variation between the types of vehicles on the road which is also likely to increase anger and aggression [10].

In addition to the gradual changes within the road network, the COVID-19 pandemic [19] has also seen sharp changes in volume and characteristics of the road fleet and in the current attitudes of drivers. In 2020 and again in 2021, the road network in Australia had a unique interruption when restrictions were placed on travel for all non-essential workers in response to the potential spread of the COVID-19 pandemic. During March and April 2020, a national lockdown mandated that people worked at home, when possible, schools and childcare facilities were closed, as were non-essential retail outlets. During a second wave in Victoria, lasting from July to August 2020 and a third wave seeing over half of the Australian population locked down again between August and October, 2021, further restrictions were placed on travel including a 5km travel limit and curfew. Thus, the characteristics of road users changed, and traffic volumes decreased significantly with reductions between 30% to almost 90% during various stages of lockdown [20,21].

International research has shown that, somewhat counter-intuitively, risky driving increased during COVID-19 lockdowns with faster speeds and more distracted driving when roads were quieter [22,23]. Reasons for this include the lack of enforcement coupled with emotional pressures placed on the drivers (e.g. economic and health concerns relating to the pandemic and associated lockdowns). Emotional pressures are a recognised trigger for anger and aggressive driving [24]. General angry mood can lead to more hostile attributions of others’ behaviours [25], which when translated into driving means angry drivers are more prone to see others as being at fault for situations that provoke anger. These evaluations can result in aggression. Likewise, research outside of road safety has shown general aggression increased during COVID-19 and periods of lockdown [26], which is likely to have also transferred to driver behaviour.

Thus, there have been acute and chronic changes to the road network in Australia, the work demands of commercial drivers and the personal circumstances for many drivers, possibly resulting in worse driving attitudes and mood. The emotional effects of the pandemic are likely to outlast the physical restrictions imposed because of it, and as traffic volumes increase to pre-pandemic levels, or beyond, it will be important to understand the implications of this for aggressive driving. These changes are likely to be linked to increases in anger and aggression while driving and in turn increased crash risk. Given the risk these behaviours pose to road safety it is important to understand if this is the case and what factors underpin these changes.

### 1.2 Most drivers feel that other drivers are becoming more aggressive, but self-report data does not support this

Despite the need to understand whether aggressive driving is increasing and design strategies around its prevention, the studies to date make any changes unclear. Researchers have shown the perceptions of aggression from other drivers have increased over time [27–29]. An international Gallop poll surveying 23 Countries, including Australia, compared self-reported prevalence of aggression received from other drivers reported in 1999 and again 2002. In Europe and Australia, a higher percentage of drivers had received aggression from other motorists in the past 12 months in 2002 compared to 1999. When asked if aggression had increased in recent years, 80% of drivers from Australia reported that it had [27]. A similar percentage of drivers in surveyed in Canada in 2006 (88%) agreed that aggressive driving had increased over the past five years [29]. A Danish study conducted in 2005 [28] asked drivers whether they had been yelled at, received a rude hand signal or been threatened by other drivers. The survey was repeated in 2016 on an independent sample. A greater percentage of drivers had been yelled at, received the finger, and threatened in 2016, when compared to the earliest timepoint of 2005. Thus, drivers feel that more drivers are becoming aggressive on the roads. However, it may be due in part to an increased number of road-users on the network.

When drivers are asked about their own aggression, and this is compared over time, there seems to be little change. However, this may be less of a reflection on aggression changes and more about how aggression is measured, the period over which it is measured and what is used to define aggression. For example, some studies provide evidence of the percentage of drivers who engage in different types of behaviours (e.g. take a population prevalence approach, [30]). Other studies compare frequency of aggression within drivers, considering not just whether it is done or not, but how often. Very few studies, ask the same driver about this frequency of aggression over time. Examples of these points are provided below.

This population prevalence approach has been used in Australia with population weighted samples (for age and gender). Comparison of findings show similar patterns across different data collection time points. Data collected on a nationally representative sample of drivers in 2014, showed that up to 70% of drivers reported minor aggressions with this measured as sounding the horn when angry in the previous two years [30]. A survey conducted in 2020 showed that less than half of those surveyed (45%) reported minor aggressions but these were measured as shouting, cursing and gesticulating and measured over a 12-month period. Thus, comparison of these results suggest that minor aggression is decreasing on roads. However, these minor aggressions differed across the two surveys making direct comparisons problematic. Likewise, results of the Gallup poll global survey conducted in late 2002 showed that 60% of drivers in Australia reported being aggressive in a 12-month period [27]. However, in this study aggression was defined as “aggressive behaviour” and while it followed several questions regarding behaviours such as changing lanes without indicating, flashing lights etc, no specific types of aggression were examined. Thus, interpretation of what is aggressive by respondents is likely to vary and if it relies on the previous questions, may represent risky aggressive behaviour.

Some researchers have been more specific in their measurements of aggression and compared the prevalence of different types of aggression over time, albeit using independent groups at each timepoint. This provides a more consistent measurement for aggression and allows for a range of behaviours to be considered (from minor to extreme aggression) and an understanding of what problematic behaviours increase over time. Using this level of sensitivity, these researchers have shown that some types of behaviours increase, while others remain stable [28,29]. Vanlaar et al. [29] surveyed drivers in Canada in 2002 and 2006 asking for frequencies of minor aggressive behaviours (swearing, sounding the horn, and gesticulating). They found very few differences in reported prevalence of these behaviours between the two time-points, with variations of +/- 2–4% only. Møller and Haustein [28] compared self-reported aggressive gesticulating over three time points, 2005, 2008 and 2016 and found that the prevalence significantly decreased between 2005 to 2008 (10% cf 7%) but increased in 2016 (13%). Additionally, they compared the prevalence of yelling, threatening other road users and hitting other vehicles or road users between 2005 and 2016 and found that yelling and hitting other vehicles had increased significantly from 12% to 19% and .3% to 1.6%, respectively, while there was no difference in the more aggressive behaviours. Therefore, international data measuring the same behaviours over time show that some aggressive behaviours increased, and these seem to be the more minor aggression, while others remain relatively consistent within the driving population. However, whether this is the case in Australia is unknown. Further, it should be cautioned that these studies used different samples across the time-points and while population weighted, compare occurrence (percentage of drivers who do this) not frequency (how often a driver does this) of only a few aggressive behaviours.

To illustrate this point, some studies provide understanding of the frequency of a driver’s aggression. In other words, how frequently each driver engages in aggressive behaviours, versus whether they have engaged in aggression or not across a specific period. This conservative consideration of aggression provides specific information about aggression on the road, which is important when designing strategies to reduce aggression. By way of example, a driver who is frequently aggressive, may need to be considered differently to a driver who has only engaged in aggression once or twice in a two-year period. Moreso, it is important to focus strategies on key road safety behaviours: i.e., a driver who frequently uses their vehicle in a risky way when angry, rather than someone who may sometimes sound their horn when angry. Data from the 2002 Gallop poll, which showed that 60% of drivers reported aggression, details specifically that 14% of drivers were aggressive “several times”, 31% of the drivers in the 2014 sample reported “occasional” aggression (the closest category to “several”) and 2% reported “frequent” use of their horn or indicating annoyance any way they could. Therefore, it may be that at a population prevalence level, aggression has not changed, but rather aggression frequency may have changed within the driver. That is, aggressive drivers may be more aggressive now, than they were previously, and the types of aggression used may have also changed.

### 1.3 Aims

The aim of this study was threefold. First, to understand whether the perceived frequency of one’s own aggression has increased (over a five-year period). Second, to understand factors associated with this increase (e.g age, gender, annual kilometres and other drivers’ aggression). Third, to explore the perceived impact of COVID-19 on driver aggression.

## 2. Materials and methods

### 2.1 Participants

A total of 774 licensed drivers who had been driving for the previous five years provided complete responses to an online survey. The socio-demographic information collected for the sample is presented in Table 1. As can be seen, mean age was 48.7 (SD = 12.8) with 256 (33.5%) women and 508 (66.5%) men. The majority (330; 42.9%) reported annual kilometres driven of more than 20,000km with 293 (38.1%) driving between 10,001 and 20,000km per year. One hundred and forty-seven drivers (19.1%) reported an annual distance of 10,000km or less per year.

**Table 1:** Socio-demographics of the sample (N = 774).

	Mean (SD) Or %
Age (years)	48.7 (13.9)
Gender	
** **Men	66.5%
** **Women	33.5%
Licence duration (years)	30.6 (14.3)
In the previous year have you been involved in a crash while you were driving?	
** **Yes	12.2%
** **No	87.8%
How many crashes have you been involved in, in the previous year?	Range 0–4 Median = 0, IQR = 0
In the previous year have you received a traffic fine or infringement notice in the past year? (not including a parking offence) (yes)	14.2%
** **Yes	14.2%
** **No	85.8%
How many infringement notices have you received in the previous year?	Range 0–10 Median = 0, IQR = 0
Estimation of average annual kilometres driven Low (0–10,000) Medium (10,001–20,000) High (20,001 or more)	19.1% 38.1% 42.9%

### 2.2 Procedure

Ethical approval was provided by the lead author’s institution. An online study was conducted between 7^th^ July and 30^th^ November 2020. The survey was hosted on Qualtrics and advertised via social media platforms (Facebook, Twitter). Upon clicking on the survey link, participants were first provided with an explanatory statement informing them about the study purpose and protocol and overview of survey items and their right to stop participating at any stage during the survey. Once they had read this, they ticked a box to confirm they had read and understood the explanatory statement (thus providing written and informed consent) and consented to take part. Inclusion criteria was being over 18 years of age and holding a valid driver’s licence. Length of licence tenure was not an exclusion criterion, however only the results for those who had been licenced at least five years were included in this study. Therefore, while an initial 920 drivers provided complete responses to the survey, data for only 774 are presented below and reported in the participants section above. The survey took approximately 10 minutes to complete, and participants were able to enter the draw to win a $100 voucher via a separate link, to protect anonymity.

### 2.3 Materials

Demographic information was sought for age (in years), postcode, gender (man, woman, non-binary/gender diverse, other), annual kilometres (categories increasing by 5,000km) and crash history. A multi-methods approach was used including validated scales (described below) and open text boxes to understand perceived changes in own behaviour and that of other drivers. This was considered across the previous five years, and also in relation to COVID-19. For questions regarding self-reported changes in aggression in the previous five years and annual mileage participants were asked to consider these before COVID-19.

#### 2.3.1 Driving anger expression inventory (DAX)

Self-reported aggression was measured using the short version of the DAX [4,31]. This is a 15-item questionnaire where participants estimate the frequency of how they express or deal with their anger when driving (1 = almost never; 4 = almost always). Aggressive expressions of anger are classified into three broad types of aggression; personal physical aggression, use of vehicle to express anger and verbal aggression. A fourth way of dealing with anger is adaptive approaches (e.g., “Tell myself it’s not worth getting mad at”). Higher scores on each factor represent more frequent expressions of that type. The short DAX has shown good reliability with Cronbach alphas ranging from 0.77 to 0.88 [31].

To understand self-reported changes in aggression, participants responded to the DAX twice: once for their current driving practices and again for their behaviour five years ago. Participants were instructed that for the same set of items, to indicate how frequently they engaged in each one five years ago. Those who had not been driving for five years were asked to skip the second presentation of the DAX. The presentation of items within both DAX scales were randomised. A total mean score of the aggressive expressions (personal physical aggression, use of vehicle to express anger and verbal aggression) for current driving and five years ago was calculated. An aggressive expression change score was generated for each participant by subtracting the current score from the five year ago score. The same process was repeated for each of the DAX’s four factors.

#### 2.3.2 Perceived change in aggression from other drivers

A single item, “Compared to FIVE years ago, would you say other drivers are: Less aggressive, about the same or more aggressive” was included to identify their perception of how aggression from other drivers has changed. Participants who indicated a belief that aggression had increased were provided with additional questions regarding the type. These conditional questions asked about four specific types of aggression from other drivers; (1) shouting, cursing or making rude gestures (to represent mild forms of aggression), (2) threatening to hurt you or others with you, (3) intentionally damaging or attempting to damage the vehicle you are in, and (4) intentionally hurting or attempting to hurt you. These items were based taken from Deffenbacher and colleagues [32].

#### 2.3.3 Perceived change in situations likely to provoke anger

All participants were asked to indicate whether they thought certain driving situations had changed over a five-year period, and how so. These included the four broad types of anger provoking situations identified in the Measure for Angry Drivers scale, which have been associated with aggressive responses [18]. These are travel delays, dangerous driving and general hostility from other drivers. An additional item: driver discourtesy was also included. A three-point response was provided for each situation (decreased, about the same or increased).

#### 2.3.4 COVID lockdown related driving changes

Participants were asked if their driving style had changed during (or due to) the COVID-19 lockdown. Any that answered yes were asked to explain how it had changed in an open text box. A similar question was asked about driving styles of other drivers during the lockdown. Anyone who answered yes was given an opportunity to describe how they thought others had changed their driving styles.

### 2.4 Data handling and analysis

A number of variables were recoded for the analysis. Annual kilometres driven was recoded into low (0–10,000kms), medium (10,001 to 20,000) and high (20,001 or higher). These categories were based on the average annual mileage for drivers in Australia (average distance travelled was 12,100km [17]). For the question of perceived changes in aggression from other drivers, responses of “less aggressive” and “about the same” were collapsed to distinguish between those who perceived an increase in others aggression and those that did not. Likewise, responses of “decreased” and “about the same” were collapsed in to distinguish between those who perceived an increase in their own aggression and those that did not.

Drivers were classified into one of two groups based on their change scores on the total DAX score (i.e. combined items from verbal, personal physical and use of vehicle). Drivers with a score of zero or less were classified as “same or better” and drivers with a score above zero were classified as “more aggressive”. Based on graphical inspection of data distributions all data was considered non-parametric. Group differences were examined using Mann-Whitney or chi-square test. The Wilcoxon matched pairs signed rank test was used to compare paired data. A binary logistic regression model was used to identify the associations of all four DAX factors with driver age, annual kilometres driven (low, medium, high), gender (only using men or women) and perceived increase of other drivers’ aggression (yes/no). Multicollinearity among the identified variables for each model was assessed using the variance inflation factor (VIF), with the highest value of 1.03 below the recommended cut-off of less than ten [33]. To avoid over-fitting the regression model, the event per variable (EPV) was calculated. The EPV is the ratio of outcome events to the number of predictor variables and is expected to be at least 10 [34]. Results for each model are reported using odds ratios (ORs) and 95% confidence intervals (CIs). The Hosmer and Lemeshow and likelihood ratio tests were used to establish how well the model fit the data [35]. Descriptive data and regression analysis were conducted with R (version 3.5.1).

An *a-priori* power analysis was conducted to determine the required sample size to detect a small effect with the logistic regression analysis (odds ratio of 1.5) at a significance level of α = .05 for a one-tailed test. The minimum required sample was 237 for maintaining a power at .80. Thus, the study was adequately powered.

**Fig 1:**
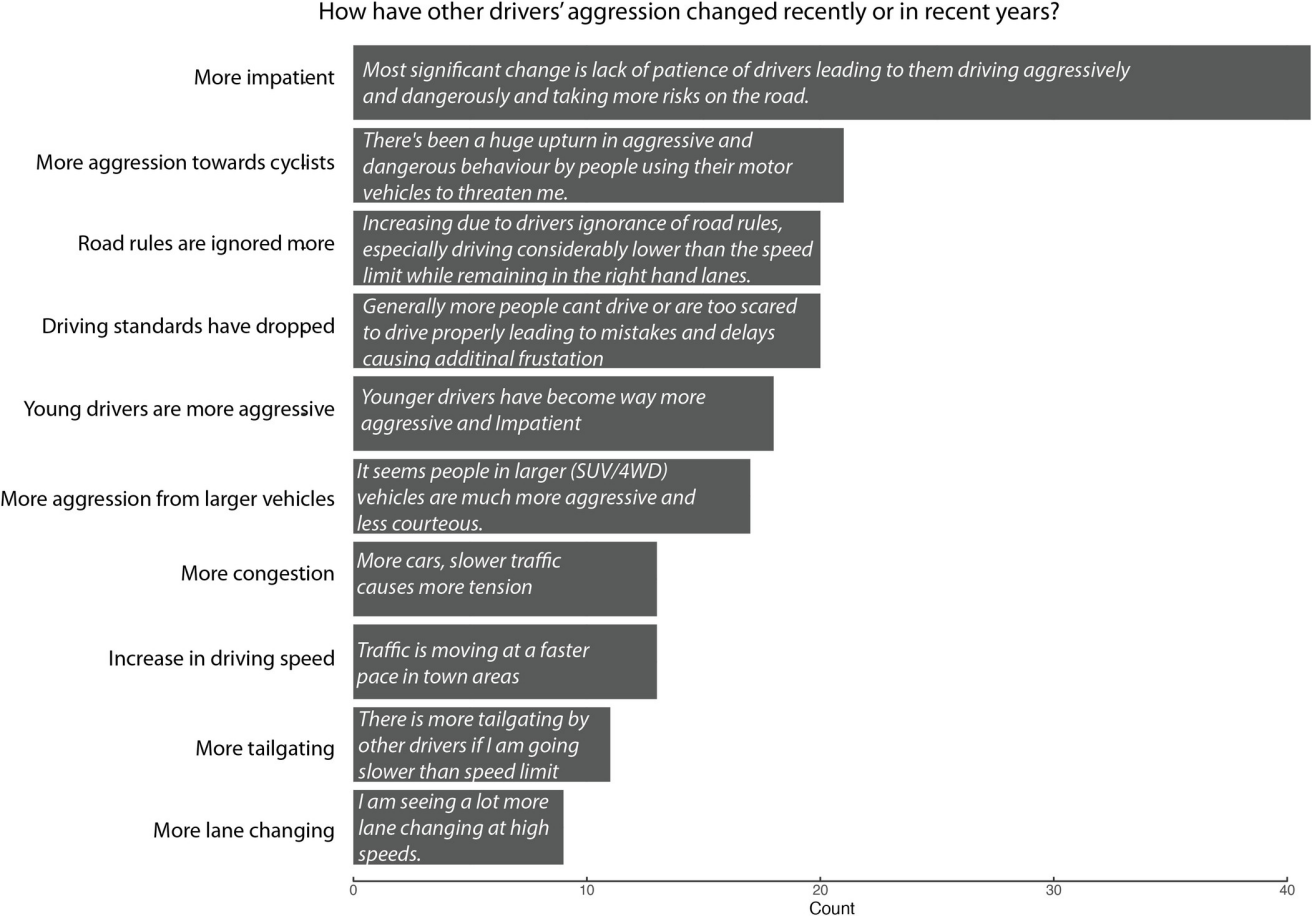
Summary of themes that emerged from coding the text responses to questions regarding changes in aggressive driving over time. No specific time frame was provided for this question. Each theme is ordered by frequency and is presented with an illustrative quote.

**Fig 2:**
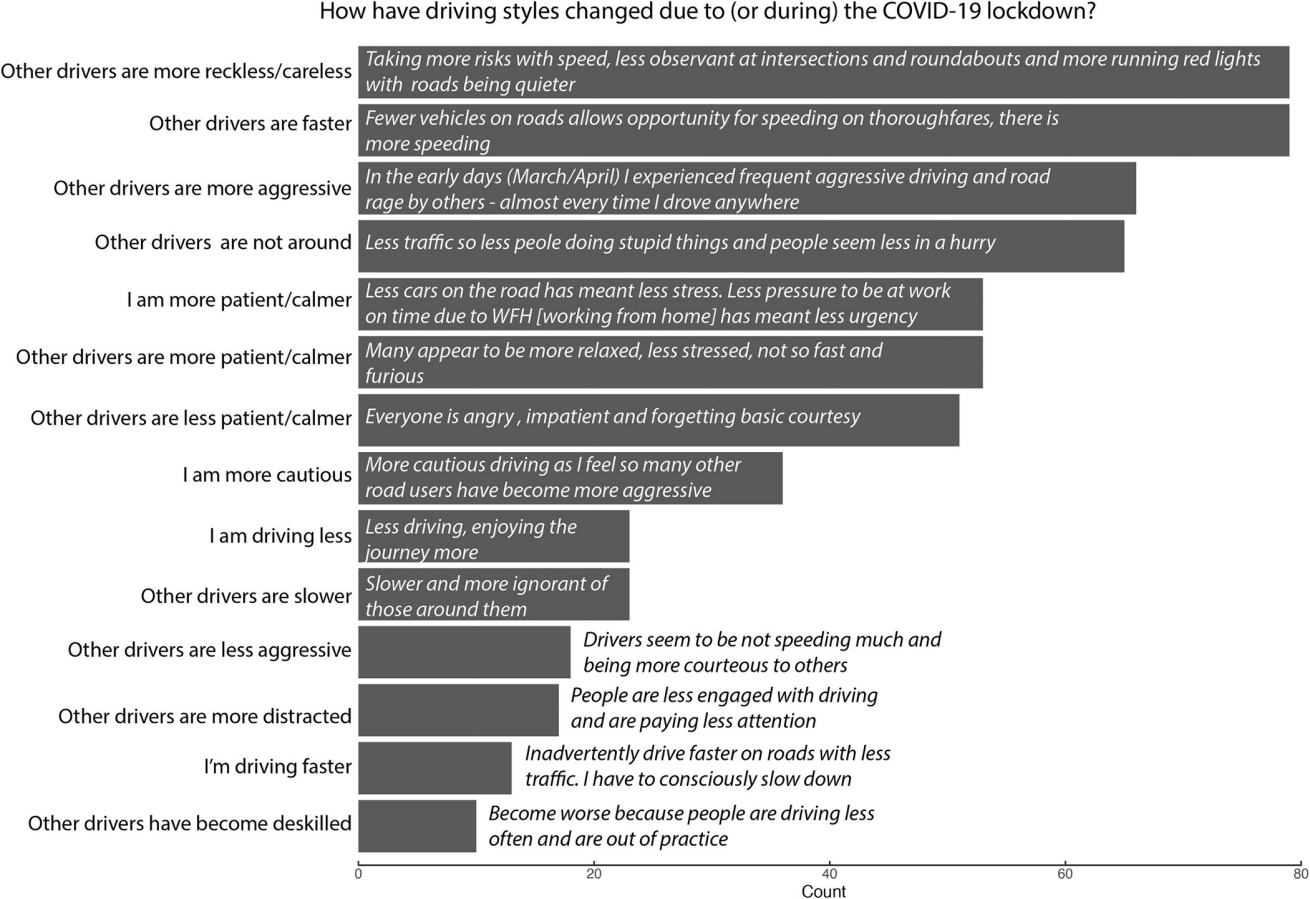
Summary of themes that emerged from coding the text responses to questions regarding changes in driving during the COVID-19 lockdown (regarding oneself and others). Each theme is ordered by frequency and is presented with an illustrative quote.

The text responses were analysed qualitatively. Not all participants provided qualitative responses. For the question asking whether drivers had any comments about aggression and whether this had changed recently, 525/774 (68%) provided text responses. For the COVID-19 related driving changes, 749 participants were driving during lockdown, 198 (26%) of them said that their own driving style had changed during (or due to) the COVID-19 lockdown and provided further explanation of how. Almost half 357/749 (48%) believed that the driving style of others had changed during this time and described this in the text box. After reviewing all text responses, co-author ST generated a list of initial themes that were discussed among authors. Codes were developed for each theme and applied to each text response. Lead author AS coded fifteen percent of all responses, which were then compared with those by co-author ST. Any discrepancies were discussed until consensus was reached. Codes were then grouped into themes by AS and ST. All themes were counted and the ten most frequent were presented visually for both general aggression (Fig 1) and COVID-19 related driving changes (Fig 2).

## 3. Results

### 3.1 Perceived change in other drivers’ aggressive expression

Sixty-one percent of participants believed other drivers were more aggressive compared to five years ago (60.8%). Nearly a third believed it was the same (32.6%) and 6.6% perceived that aggression had decreased.

Those who reported that aggression had gotten worse (n = 471) were asked four follow-up questions regarding the type/s of aggression that had changed. Drivers in other vehicles shouting, cursing or making rude gestures was the most common type, with 77.8% indicating that this had increased. In comparison, a lower percentage of respondents reported perceived increases in the other type of behaviours. For example, 38.5% reported increases in perceived physical threats, 34.5% reported increases in attempts to or actual damage to vehicle and 27.6% reported attempts to or actual physical harm.

In addition to perceived increases in aggression from other drivers, situations that are likely to provoke anger were also largely seen as increasing. For example, most participants reported that there had been an increase over the past five years in discourtesy from other drivers (58.6%), dangerous driving (66.5%), general driver hostility (56.0%) and travel delays (66.3%).

### 3.2 Perceived change in own aggressive driving

Table 2 shows the mean scores for the perceptions of aggressive driving now, and previously. Cronbach alphas (*α)* for each subscale are also presented. All subscales showed acceptable reliability with alphas ranging between .66 and .89. Using the paired Wilcoxon signed ranked test, significant increases in self-reported adaptive constructive ways of dealing with anger between now and five years ago were found (p < .001). A statistically significant reduction was observed for perceived personal physical aggression (p < .001) and use of vehicle to express anger (p < .001) compared to five years ago. However, no change in verbal aggressive behaviour was evidenced (p = .146).

**Table 2:** Means and SD of driving anger expression items now and five years ago (n = 774).

		Five years ago M (SD)		Now M (SD)	
*Factor 1*: *Adaptive/Constructive*		*2*.*60 (0*.*83)* *α = 0*.*89*		*2*.*76 (0*.*71)* *α = 0*.*80*	
*Item*		*M*	*SD*	*M*	*SD*
7	Think of positive solutions to deal with the situation	2.33	1.01	2.41	0.99
10	Tell myself it’s not worth getting mad at	2.62	0.99	2.78	0.99
11	Tell myself it’s not worth getting involved	2.69	1.02	2.89	1.00
14	Accept there are frustrating situations	2.69	0.95	2.90	0.86
15	Tell myself to ignore it	2.66	0.98	2.80	0.95
*Factor 2*: *Personal physical aggressive expression*		*1*.*18 (0*.*35)* *α = 0*.*66*		*1*.*14 (0*.*32)* *α = 0*.*71*	
*Items*		*M*	*SD*	*M*	*SD*
3	Try get out of the car and tell the other driver off	1.09	0.37	1.06	0.31
4	Roll down the window to communicate my anger	1.37	0.67	1.27	0.56
5	Try to scare the other driver	1.22	0.56	1.16	0.46
13	Try to get out and have a physical fight	1.05	0.29	1.06	0.34
*Factor 3*: *Verbal aggressive expression*		*2*.*06 (0*.*78)* *α = 0*.*83*		*2*.*03 (0*.*73)* *α = 0*.*77*	
*Items*		*M*	*SD*	*M*	*SD*
2	Make negative comments about the driver aloud	2.31	0.90	2.43	0.89
9	Swear at the other driver aloud	2.11	0.94	2.11	0.94
12	Yell at the other driver	1.76	0.87	1.61	0.81
*Factor 4*: *Use of Vehicle to express anger*		*1*.*47 (0*.*59)* *α = 0*.*75*		*1*.*39 (0*.*52)* *α = 0*.*68*	
		*M*	*SD*	*M*	*SD*
1	Drive right up on the other driver’s bumper	1.41	0.68	1.33	0.62
8	Drive a lot faster	1.61	0.78	1.54	0.75
6	Do to drivers what they did to me	1.39	0.69	1.31	0.61

*α* = Cronbach’s alpha.

Although at a group level the DAX factor scores showed a slight decrease in personal physical aggression and use of the vehicle to show anger, Table 3, shows that one third of participants (33.3%) believed that their own aggression when driving had increased over the previous five years. Sixty six percent believed their aggression was the same now (24.7%) or less frequent (42.1%).

**Table 3:** Distribution of self-reported changes across different types of aggression (N = 774).

Aggressive behaviour	Less frequent %	Same frequency %	More frequent %
Adaptive constructive ways of dealing with anger	30.0%	20.3%	49.7%
Verbal aggression	34.9%	35.1%	30.0%
Aggressive use of vehicle	25.4%	57.5%	17.1%
Personal physical aggression	22.1%	66.5%	11.4%
Total aggression (does not include adaptive)	24.7%	42.1%	33.3%

Table 4 shows that there were no significant differences in age, gender, licence duration, crash involvement or mileage between drivers who believed their aggression had increased, compared to drivers who believed their aggression was the same or lower. However, a relationship was found between increases in aggression and increases in perceived aggression from other drivers. Likewise, drivers who felt they had become more aggressive also reported that situations of driver discourtesy, dangerous driving and general hostility from other drivers had increased. Interestingly, there was no difference between groups on perceptions of travel delays, suggesting that drivers who thought they had increased their aggression were no more likely than those who had not to perceive travel delays had increased.

**Table 4:** Demographic differences between drivers whose aggression had increased, versus those whose aggression is the same or lower (N = 774).

Demographic and driving characteristics	Perceived own driving anger expression: compared to five years ago		
	Same or lower (66.7%; n = 517)	Higher (33.3%; n = 258)	P value*
	Mean (SD) Or %	Mean (SD) Or %	
Age (years)	49.0 (13.7)	48.2 (14.2)	.465
Gender			
** **Men	67.1	65.2	.657
** **Women	32.8	34.8	
Licence duration (years)	31.6 (14.3)	29.9 (14.3)	.331
In the previous year have you been involved in a crash while you were driving? (yes)	12.8%	10.9%	.520
How many crashes have you been involved in, in the previous year? Range 0–4, Median = 0; IQR = 0	0.18 (0.55)	0.15 (0.48)	.495
In the previous year have you received a traffic fine or infringement notice in the past year? (not including a parking offence) (yes)	14.5%	13.6%	.831
How many infringement notices have you received in the previous year? Range 0–10; Median = 0; IQR = 0	0.19 (0.71)	0.14 (0.49)	.583
Estimation of average annual kilometres driven Low (0–10,000) Medium (10,001–20,000) High (20,001 or more)	21.4% 37.0% 41.6%	14.5% 40.2% 45.3%	.069
Perceived aggression from other drivers compared to five years ago Lower Same Higher	7.9% 36.0% 56.1%	3.9% 25.7% 70.4%	< .001*
Travel delays compared to five years ago Lower Same Higher	4.5% 29.1% 66.5%	3.2% 31.1% 65.8%	.612
Driver discourtesy compared to five years ago Lower Same Higher	8.0% 36.8% 55.2%	5.1% 29.4% 65.5%	.021*
Dangerous driving compared to five years ago Lower Same Higher	5.0% 31.9% 63.1%	3.5% 23.1% 73.4%	.016*
General hostility compared to five years ago Lower Same Higher	8.3% 40.0% 51.6%	3.9% 31.3% 64.8%	< .001*

*Significant p values < .05 Based on Chi-Square test of difference and between group t-tests.

Logistic regressions were conducted to better understand the associations between driver characteristics and perceived changes on each of the four DAX variables (see Table 5). All variables were retained in the final models to act as controls. Of the three aggressive expression factors, only the perception that other drivers have become more aggressive was associated with the belief one’s own aggression had increased. Overall evaluation of these models was good with acceptable goodness-of-fit statistics. The model examining change in adaptative expression of aggression was not satisfactory in either improvement over the intercept only model or goodness-of-fit. Accurate model estimation was indicated by the EPV being above ten (225/5).

**Table 5:** Crude and adjusted odds ratio (OR) for variables associated with change in own driving anger expression based on the four types of aggressive expression.

	*Adaptive/Constructive*				*Personal physical aggressive expression*				*Verbal aggressive expression*				*Use of vehicle to express anger*			
*Covariate*	Crude OR (95%CI)	P	Adj OR (95%CI)	P	Crude OR (95%CI)	P	Adj OR (95%CI)	P	Crude OR (95%CI)	P	Adj OR (95%CI)	P	Crude OR (95%CI)	P	Adj OR (95%CI)	P
Gender, (men referent)																
Women	0.95 (0.70,1.29)	.738	0.90 (0.66,1.22)	.433	0.98 (0.60,1.58)	.921	1.00 (0.61,1.63)	.988	0.89 (0.64,1.24)	.479	0.85 (0.6,1.19)	.340	**1.65 (1.12,2.43)**	**.011** *	**1.70 (1.14,2.55)**	**.033** *
Age (years)	0.99 (0.98,1.00)	.063	**0.99 (0.98,1.00**)	**.047** *	1.00 (0.98,1.02)	.693	1.00 (0.98,1.01)	.955	1.00 (1.00,1.01)	.913	1.00 (0.98,1.00)	.713	**0.98 (0.97,0.99)**	**.007** *	**0.98 (0.97,0.99)**	**.007** *
Annual KMs	-	-														
Low (0–10000)																
Medium (10001–20000)	0.86 (0.57,1.29)	.461	0.86 (0.62,1.18)	.461	1.86 (0.86,4.02)	.115	1.83 (0.84,3.99)	.126	1.54 (0.97,2.45)	.070	1.24 (0.87,1.76)	.227	**2.73 (1.41,5.28)**	**.003** *	**3.15 (1.61,6.17)**	**< .001** *
High (20001 +)	0.79 (0.52,1.18)	.203	0.77 (0.52,1.15)	.203	**2.44 (1.16,5.13)**	**.019** *	**2.31 (1.09,4.91)**	**.029**	**1.83 (1.30,2.54)**	**.017**	1.19 (0.72,1.97)	.495	**2.62 (1.37,5.03)**	**.004** *	**2.93 (1.51,5.71)**	**.002** *
Aggression from other drivers (5years)																
Lower or the same	-	-														
Higher	1.07 (0.79,1.43)	.669	1.13 (0.84,1.53)	.420	**2.1 (1.25,3.52)**	**.005** *	**2.03 (1.21,3.42)**	**.008** *	**1.82 (1.30,2.57)**	**< .001** *	**1.83 (1.31,2.58)**	**< .001** *	**1.61 (1.07,2.42)**	**.022** *	**1.61 (1.05,2.45)**	**.028** *
Overall model			χ^2^ (df)	p			χ^2^ (df)	p			χ^2^ (df)	p			χ^2^ (df)	p
Likelihood ratio test			5.90(5)	.317			14.26(5)	.014*			18.53(5)	.002*			33.361(5)	< .001*

NB: Hosmer & Lemeshow test across all models were non-significant p > .05; Bold demonstrates significant Odds Ratios

* significant p values < .05.

As can be seen in Table 5, the main factor associated with perceived increases in aggression was the perception that other drivers were also more aggressive. This was significantly associated with increased personal physical aggression, verbal aggression and use of the vehicle to express anger and when age, gender and annual mileage were controlled for. Drivers who believed other drivers were more aggressive had almost twice the odds of also reporting increases in their own aggression across all different types of aggressive expression of anger.

### Qualitative analysis of open text boxes

Fig 1 shows the general themes in response to the question seeking comments about aggression and how this has changed. Overall, drivers discussed other drivers. They felt other drivers were now more impatient and displayed more aggression toward cyclists and ignored road rules.

Fig 2 shows the responses to how one’s own and others’ driving styles have changed during COVID-19 related lockdown. Interestingly, the most common themes were an observation that drivers were taking more risks, driving faster, and exhibiting more aggression. Similarly, 525 participants provided a text response to the question about driving aggression and whether this has changed recently. A common theme was that drivers had become more impatient, more aggressive towards cyclists and less observant of road rules.

## 4. Discussion

The aim of the current study was to understand whether drivers report changes in aggression over time, and what types of change. To do this, drivers self-reflected on their own verbal aggression, aggressive use of the vehicle and personal physical aggression, providing retrospective frequencies for each for five years ago as well as reporting current tendencies (with current being before the COVID-19 lockdowns). Our results showed that one third of drivers believed they were more aggressive now, than five years ago, while most felt that their aggression had either not changed (25%) or they were less aggressive (42%). Almost half of the sample believed (49.7%) that they more frequently try to deal with anger in an adaptive manner now compared to five years ago, suggesting that overall, most drivers feel they have become less aggressive over time.

A further aim of the study was to understand what factors were associated with self-reported increases in aggressive driving. This is important given that one-third of drivers felt they were now more aggressive than they were five years ago. These potential increases in aggression are likely to be linked to increased crash risk [1]. We found that the factors associated with increases in self-reported aggression were being younger, being a woman, driving longer distances and believing that other drivers are also now more aggressive. Higher mileage and perceiving others as having become more aggressive were significant across all types of aggression, while age and gender were only associated with increases in use of the vehicle when angry. This finding appears to contradict previous research showing that age and gender have been found to be related to all types of aggression [4–5,30,31,36]. That, younger, male drivers tend to report higher levels of all three types of aggression [4,37]. However, our lack of associations between these factors and change may be explained by the older age of the sample (average age 49), with aggression being more prevalent in young people, particularly under 26 [38] and drivers under 39 [30]. Further, that a greater percentage of women reported increasing their risky vehicle use when angry, compared to men may represent some sense of leveling out, whereby women become more aggressive (or believe they do) as they get more experienced, but this does not mean they have higher levels of this type of aggression compared to men. To support this point, mean comparisons between scores on the use of the vehicle when angry factor showed no significant mean differences across men and women for current levels of aggression (p = .67).

A significant factor associated with perceived increased aggression was the view that others had also become more aggressive. This finding is noteworthy for two reasons. First, it is in line with social norm theory, that the perception of others’ behaviours guides our own [39]. Social norms are the perceptions of what is normal or acceptable in a specific situation. These cover the perception of what is approved of by others, or the belief of what is commonly done by others in a situation [40]. The idea that a person may have internalised standards which can influence their behaviour, based on what they think others do, has been utilised by road safety researchers [41]. A wide range of road safety behaviours have been associated with social norms, from intention to speed [42], alcohol consumption when driving [43] and aggressive driving behaviour towards cyclists [44]. Indeed, it may be the perception of others’ aggression, rather than one’s own aggression that is an important risk factor in crashes [36]. This research emphasizes that perception (not the reality) of other drivers’ behaviours is important to consider when attempting to explain driver behaviour. Thus, our findings also support the hypothesis that drivers may develop more aggressive driving styles when they believe the driving environment is more aggressive, and this in turn may increase their crash risk.

The qualitative findings support this. Drivers felt that other drivers had less patience than previously, were now more aggressive towards cyclists and had worse driving behaviour (i.e. rule violations and lower standards of behaviour). The COVID-19 lockdowns seemed to exacerbate these poorer behaviours, despite there being less traffic on the road, and thus less potential for many identified anger provoking situations (i.e., travel delays, hostile interactions with other drivers; [18]). This may represent a continuation of existing social norms, that remain in the face of dramatic changes in road environment. Thus, efforts to reduce aggression need to focus on challenging driver attitudes about the general driving culture.

Second, while peripheral to the main aims, our findings also showed relationships between feeling that one’s aggression had increased and the belief that situations likely to provoke anger had also increased. Interestingly, there was a higher percentage of drivers who felt they were more aggressive now than before also reporting that driver discourtesy, dangerous driving and general hostility from other drivers had increased. However, there was no relationship between changes in aggression and the perception that there were more travel delays now compared to five years ago. Taken together, these findings suggest that congestion may not be a factor in perceived driving aggression increases. Rather, it may be the perceptions of other drivers, or perhaps more broadly perceptions that the driving culture is getting more aggressive, that is related to the belief that one’s own aggression is increasing. The finding that 61% of drivers in the current study said they think other drivers are now more aggressive than five years ago, suggests that there is an overall perception that driving is becoming more aggressive.

A final aim of the study was to understand how acute changes in traffic volume related to COVID-19 lockdowns influenced perceived aggression. This provided a unique opportunity to study aggression during decreased traffic volumes, but potentially increased emotional pressure. As mentioned above, the main theme that emerged was that other drivers were still seen as more reckless, aggressive and driving faster, with almost half of those driving during lockdowns reporting this. This provides further support for our suggestion above that driving aggression is less associated with congestion and more associated with attitudes and perceptions of other drivers. As we emerge out of COVID-19 lockdowns, research needs to focus more on the longer-term impact on drivers. Key focus could be on emotional and cultural changes influencing poorer driving. For example, understanding further how chronic negative mood or stress [25] influences decisions and evaluation made while driving; and supporting more positive perceptions and interactions with other road users including drivers and cyclists.

Following from this, our findings suggest that strategies to support drivers recognise and resist aggression could focus on understanding the driving culture and perceived social norms within it. Indeed, aggression can be context specific, with social boundaries for what is acceptable differing across situations (e.g. [45]). This may be why gender may not always be related to aggression in a driving context or related to different types of aggression [46] because the situational norms of driving may dictate what responses are appropriate and these may differ from other contexts. Qualitative data in the study suggest that overall, the driving culture is seen as more impatient, less tolerant of cyclists and overall, other drivers are seen as more dangerous and less skilled. These are areas to focus on when developing strategies to reduce anger and aggression.

### Limitations

The methods are limited by perceived social desirability bias and the retrospective method for understanding changes in behaviour. For example, it may be possible that drivers are biased to under-reporting current behaviour but report previous behaviour more accurately. Likewise, given the presentation order of the aggression questionnaires (current levels sought before previous levels) information on previous behaviour may be influenced by responses given for current behaviour. While every effort was taken to reduce socially desirable responding overall (by the anonymous online nature of the data collection; [47]), this potential bias must still be recognised as a limitation of the current study. This limitation could be overcome in future studies by randomising the order of aggression surveys or ensure they were separated by other survey items to mitigate order effects. The retrospective nature of the measurement for aggression five years ago, also limits the findings to perceptions of change within the driver. Although in terms of social norms, the emphasis on perception of change in others is perfectly reasonable. However, this information cannot be assumed to represent actual change. While there is some evidence to show relationships between retrospective and prospective measurements for behavioural frequency outside of driving [48], none exists within the driving literature. Longitudinal self-reported studies are needed to understand changes in behaviour over time using the same participants and these could also include a retrospective and prospective component to further understand the effectiveness of aggressive behaviour recall.

There may be also other factors associated with changes in aggression that were not measured in the current study. For example, sensation seeking [49,50] has been shown to be related to riskier behaviour and faster driving speeds. However, this can have differential influences on men compared to women and across different levels of driving experience [42]. Thus, as experience increases the influence of sensation seeking on driver behaviour may change, leading to less risky behaviour. However, it should be noted that all the drivers included in the analyses had at least five years of driving experience and as a sample had held their licence for an average of 31 years. This is less likely to be an issue for this sample, but worth exploration in future studies. Likewise, alcohol or substance consumption have been found to be predictors of repeat offenders for risky driving behaviour [51,52]. This is also worth consideration in future studies. Further, this study did not measure behaviour objectively. Drivers may not have accurate perceptions of their own aggression [53]. Thus, further studies should support self-reported data with objective driving behaviour.

### Conclusions and practical implications

Our results highlight the reciprocal nature of perceived and expressed aggression. Campaigns to reduce aggressive driving, therefore need to focus on challenging beliefs that drivers are becoming more aggressive, and improving interactions, or perceived interactions among road users. This will be especially important as the COVID-19 pandemic and associated emotional burdens continue, placing drivers at higher risk of being angry or aggressive on the roads. Research on other dangerous driving behaviours such as speeding and driving when under the influence has shown similar social contexts underlying behaviour [54,55]. That is, drivers tend to report engaging in these when they perceive low risk of enforcement. This may explain why in the current study, other drivers were perceived to be driving more dangerously and aggressively during lockdown when enforcement may have been seen to be lower. Previous research has also shown that dangerous behaviours are more frequent when socially acceptable. Drivers report speeding and drink driving more frequently when they have friends and family who also engage in these behaviours [54,55]. While the evidence that targeting social norms can improve road safety behaviours is limited [56] there is a large body of evidence detailing its use more broadly [57]. As such, targeting social norms, and the aggressive driving culture may be an important strategy to reduce aggression.
